# *Lactobacillus paracasei* subsp. *paracasei* NTU 101 lyophilized powder improves loperamide-induced constipation in rats

**DOI:** 10.1016/j.heliyon.2020.e03804

**Published:** 2020-04-21

**Authors:** Chien-Li Chen, Sih-Han Chao, Tzu-Ming Pan

**Affiliations:** a Department of Research and Development Division, SunWay Biotech Co., Ltd., Taipei, Taiwan; b Department of Biochemical Science and Technology, College of Life Science, National Taiwan University, Taipei, Taiwan

**Keywords:** Food science, Microbiology, *Lactobacillus paracasei* subsp. *paracasei* NTU 101, Constipation, Rats, Fecal water content, Defecation frequency

## Abstract

Constipation is a condition of the digestive system characterized by formation of hard feces that are difficult to eliminate. It has emerged as a new problem that is commonly encountered by many people and lifestyle changes have been unsuccessful in providing a solution. This study aimed to investigate the effects of *Lactobacillus paracasei* subsp. *paracasei* NTU 101 on loperamide-induced constipated rats and on gastrointestinal tract function. Sprague-Dawley rats were administered loperamide (2 mg/kg BW) twice daily as well as 1.3, 2.6, and 13.0 mg/kg BW/rat/d of NTU 101 powder. The control, positive control, and NTU 101 powder groups (0.5, 1, 5×) showed improved intestinal mobility with a statistically significant increase of 12.4%, 14.7%, 12.5%, 13.4%, and 15.1%, respectively (p < 0.05); the fecal water content was also significantly increased by 11.7%, 9.0%, 10.0%, 9.3%, and 11.0%, respectively (p < 0.05), compared to the loperamide group. Furthermore, NTU 101 increased the *Bifidobactrium* spp. and decreased the *Clostridium perfringens* content in feces; it increased short-chain fatty acid levels, reduced fecal pH value, enhanced the thickness of the colonic mucosa, and increased the number of mucin-producing goblet cells and interstitial cells of Cajal. Thus, NTU 101 powder was found to alleviate loperamide-induced constipation and improve gastrointestinal tract function.

## 1 Introduction

Constipation is one of the most common gastrointestinal conditions worldwide, with a reported prevalence ranging from 2% to 30%, especially among old people (Dimidi et al., 2014; Koebnick et al., 2003). Constipation is also a risk factor for colorectal cancer, irritable bowel syndrome, and other gastrointestinal disorders (Shimotoyodome et al., 2000a). However, the pathogenesis of constipation is unclear and is believed to be multifactorial (Schuster et al., 2015). Lifestyle, eating habits, metabolic conditions, and neurological disorders potentially cause constipation. Because stools are retained in the large intestine for long durations during constipation, fecal water content is absorbed, which reduces bowel movements and induces bowel pain (Chen et al., 2010) and intestinal imbalance (Khalif et al., 2005). Constipation can be categorized as primary and secondary. Primary constipation can be divided into three types: (a) normal-transit constipation, (b) slow-transit constipation, and (c) defecatory disorders (Lembo and Camilleri, 2003). Management of constipation involves advising patients to exercise and consume more fluids and fiber, as addition of dietary fiber increases fecal mass and colonic transit time (Yang et al., 2012). Thus far, laxatives are most common treatments for constipation; however, they do show side effects. The most common side effects of laxatives include diarrhea, upset stomach, vomiting, and stomach cramping. In particular, osmotic laxatives containing poorly absorbable ions such as magnesium or phosphate can cause metabolic disturbances particularly in the presence of renal impairment (Xing and Soffer, 2001). Additionally, loperamide—the most commonly used antidiarrheal medication—is also used to induce constipation in animal models. However, it decreases intestinal peristalsis, fecal water content (Shimotoyodome et al., 2000b), and fecal short-chain fatty acid (SCFA) levels (Li et al., 2015). Clinical study for patients with constipation measure stool consistency, stool weight, gut transit time (whole and regional), other gastrointestinal symptoms (e.g., bloating, pain), and adverse effects/compliance. A new therapeutic approach for constipation could be based on the modulation of intestinal microflora by administering prebiotics and/or probiotics.

Recent increasing evidence indicates that probiotics can reduce constipation by improving defecation frequency and intestinal motility (Koebnick et al., 2003; Krammer et al., 2011; Magro et al., 2014). Probiotics are defined as “living micro-organisms which, upon ingestion in certain numbers, exert health benefits beyond inherent basic nutrition” (Guarner and Schaafsma, 1998). The most common types of microorganisms used as probiotics are lactic acid bacteria (LAB) such as *Lactobacillus* sp., *Bifidobacterium* sp., and *Enterococcus* sp. (Klein et al., 1998). *Lactobacillus* sp., *Bifidobacterium* sp., *Saccharomyces boulardii*, and other microbes are used as probiotics, i.e., live microorganisms used as food supplements owing to their health benefits (Fijan, 2014). Probiotics act on the large intestine by influencing the intestinal flora as well as on other organs by modulating immunological parameters and intestinal permeability, and by producing bioactive or regulatory metabolites (Markowiak and Śliżewska, 2017).

Most studies have reported that probiotic strains can survive and transit through the acidic gastric environment and through bile and pancreatic juice in the upper duodenum to exert beneficial effects in the jejunum, ileum, and colon. Convincing data regarding beneficial immunological effects were also obtained from dead cells (Mottet and Michetti, 2005). Probiotic bacteria predominantly colonize the colon, but might also have beneficial effects on the small intestine, as well as systemic effects via the immune system (Hemarajata and Versalovic, 2013). These phenomena are thought to mediate most beneficial effects, including a reduction in the incidence and severity of constipation and diarrhea, which is one of the most widely recognized uses of probiotics (Hemarajata and Versalovic, 2013). In previous studies, laxative effects of probiotics were established in rat models of loperamide-induced constipation, using sodium picosulfate as a positive control (Eor et al., 2019).

*Lactobacillus paracasei* subsp. *paracase*i NTU 101 (NTU 101) is isolated from the intestinal microbial flora of breast-fed infants in 3 days after birth (Lin et al., 2004). These aforementioned findings suggest that the intake of the cultured probiotic NTU 101 might have various beneficial effects on human and animal health. Therefore, the present study aimed to investigate the effects of NTU 101 on loperamide-induced constipated rats and on gastrointestinal tract function.

## 2 Materials and methods

### 2.1 Preparation of probiotics

*Lactobacillus paracasei* subsp. *paracasei* NTU 101 strain (commercial name Vigiis 101) lyophilized powder was used in the current study that was provided from SunWay Biotech Co., Ltd., Taipei, Taiwan.

### 2.2 Design of animal experiments

Forty-eight (8-week old) male Sprague-Dawley rats weighing 300–320 g were purchased from BioLASCO Taiwan Co., Ltd. (Ilan, Taiwan). Animals were maintained in 24 cages (2 animals/cage, n = 8/group), in an environment with a relative humidity of 50–60%, temperature of 25 ± 2 °C, and a light/dark cycle of 12 h (illumination between 0700–1900 h). All animals received humane care in according to the guidelines by the Institutional Animal Care and Use Committee (IACUC) of National Taiwan University (Taiwan, ROC). In this study, we received ethical approval for the experiment. (IACUC proof document NTU-102-EL-80). To assess the effect of probiotics on loperamide-induced constipation in the rat model, animals were divided into six experimental groups. Rats were subcutaneously injected with 2 mg/kg body weight of loperamide (Sigma-Aldrich, St. Louis, MO, USA) suspended in 0.9% saline twice daily for 20 d. Simultaneously, the control group received 0.9% saline subcutaneously. The samples were dissolved in H_2_O, and the feeding volume was 1 mL. The treatments included a control group (a group), a loperamide-induced constipation group with no probiotic treatment (b group), a loperamide-induced constipation positive control group (c group, 0.52 mg/kg sodium picosulfate; Sato Pharmaceutical, Tokyo, Japan), loperamide-induced constipation 0.5× NTU 101 group (d group, 0.5× NTU 101 (2.3 × 10^9^ colony-forming units (CFU)/kg body weight (BW)/d NTU 101 powder), loperamide-induced constipation 1.0× NTU 101 group (e group, 1.0.× NTU 101 4.5 × 10^9^ colony-forming units (CFU)/kg body weight (BW)/d NTU 101 powder), loperamide-induced constipation 5.0× NTU 101 group (f group, 5.0× NTU 101 (f, 2.3 × 10^10^ colony-forming units (CFU)/kg body weight (BW)/d NTU 101 powder). Constipation was induced as described by Chen et al. with minor modifications (Chen et al., 2010).

### 2.3 Fecal parameters

Fecal pellets, total fecal weight, fecal dry weight, fecal water content, and fecal pH values were analyzed. Each rat fecal sample was collected once daily (24 h) during the adaptation and experimental period. Fecal dry weight was obtained as reported previously (Chen et al., 2010) with minor modifications. Harvested feces were immediately weighed and dried at 60 °C for 24 h. Fecal pH values were analyzed using a pH meter (Jenco, San Diego, CA, USA). Fecal water content was calculated as Eq. (1):

(1) Fecal water content (%) = (fecal total weight - dry weight/total weight)

### 2.4 Gastrointestinal transit ratio

Charcoal meal test is widely used for the measurement of gastrointestinal transit in small rodents. At the end of experimental day, first the rats were fasted for 18 h and then fed a sample (sodium picosulfate/NTU 101). After 10 min, animals were fed an activated charcoal meal (10% active charcoal and 5% carboxymethyl cellulose). Wait at least 30 min of peristalsis and then were euthanized and the stomach and intestine were co-harvested to observe the transit distance of activated charcoal. Activated charcoal transit ratio was calculated as Eq. (2):

(2) Activated charcoal transit ratio (%) = (charcoal marker/intestinal length) × 100%

### 2.5 Intestinal microflora

Indicator bacteria were *Bifidobacterium* spp. and *Clostridium perfringens*; the latter is considered an indicator of fecal contamination*.* To obtain fresh feces, rat abdomens were massaged and then feces were immediately harvested in a centrifuge tube and treated with CO_2_ to maintain anaerobic conditions. Selective medium BIM-25 (*Bifidobacterium* iodoacetate medium 25) was used to culture *Bifidobacterium* spp. and tryptose sulfate cycloserine medium was used to culture *C. perfringens* in an anaerobic environment at 37 °C for 3 days (O'Sullivan, 2000).

### 2.6 Analysis of fecal SCFAs

We employed the method described by Shimoyama et al. with minor modifications (Shimoyama et al., 2015). In a centrifuge tube, 500 μL of 1 N HCl and 25 μL of 100 mmol/L 2-methyl valeric acid were added to 0.5 g of feces and subsequently vortex-mixed to volatilize the diethyl ether. After centrifugation (6800 × *g*, 5 min, 4 °C), 1 mL diethyl ether solution was collected. For analysis, the sample was injected into a gas chromatography-flame ionization detector equipped with an HP-INNOWAXax GC column (Agilent, Santa Clara, CA, USA). Detection conditions were as follows: initial and final column oven at 120 °C for 1 min and 200 °C for 5 min, respectively, injection and detection column temperature was 250 °C, and the flow rate was 1.85 mL/min.

### 2.7 Histological analysis of the distal colon

Histologic samples were obtained for determination of goblet cells and colonic mucosal thickness. These samples were taken from forty-eight rats divided into 6 groups (n = 8 each). Tissue specimens of the distal colon and specimens with feces inside were used for histological analysis. The thickness of the mucosal layer and the number of goblet cells were assessed). Tissue sections were stained with Alcian Blue (pH 2.5). Six random microscopic fields were selected to determine mucosal layer thickness and goblet cell number. To evaluate the inflammatory response of the colon, tissue specimens were stained with hematoxylin and eosin (H&E) and subsequently assessed by a histopathologist using a microscope.

### 2.8 Immunohistochemistry

Tissue sections were dewaxed and treated with 3% H_2_O_2_ for 15 min to block endogenous peroxidase activity, followed by treatment with 1% bovine serum albumin (BSA) for 30 min and subsequent addition of primary antibody (c-Kit polyclonal antibody; 1: 100; Bioss Inc., Woburn, MA, USA) at 4 °C overnight. Thereafter, sections were probed with a horseradish peroxidase (HRP)-labeled secondary antibody (amplifier for mouse and rabbit) for 15 min and treated with an HRP polymer detector for 15 min, followed by treatment with 3, 3-diaminobenzidine (DAB) buffer for 20 min. The tissue sections were then stained with H&E for 7 min (Brajkovic et al., 2017).

### 2.9 Statistical analysis

Data are expressed as the mean ± standard deviation (SD). Statistical significance of the differences between samples was determined by one-way analysis of variance (ANOVA) using a general linear model with SPSS version 10.0 software (IBM, Armonk, NY, USA), followed by one-way ANOVA with a Newman-Keuls post-hoc test. *P* values <0.05 were considered statistically significant.

## 3 Results

### 3.1 Fecal parameters

To investigate the effect of NTU 101 on constipation, fecal parameters were measured. Fecal water content and number of pellets are determinants of constipation. As shown in Table 1, fecal pellet numbers, fecal total weight, and fecal water content of the loperamide group were significantly lower than in the control group (*p* < 0.05); however, these parameters were significantly higher in the positive control and NTU 101 (0.5, 1, 5×) treatment groups than in the loperamide group (*p* < 0.05). Compared with the loperamide group, fecal pH values were significantly lower in the control (*p* < 0.05) and NTU 101 (0.5, 1, 5×) treatment groups (*p* < 0.05). Table 1 also shows that the number of fecal pellets in the colon was significantly higher in the control and NTU 101 (0.5, 1, 5×) treatment groups than in the loperamide group (p < 0.05), similar to the positive control group (p < 0.05).

**Table 1:** Fecal parameters of rats from day 15 to day 20.

Groups	Pellet numbers	Total weight	Dry weight	Water content	pH value
	(n/day/rat)	(g/day/rat)	(g/day/rat)	(%/day/rat)	D 20
Control	68.18 ± 1.29	14.83 ± 0.36	7.68 ± 0.31	48.21 ± 0.14	5.85 ± 0.05
Loperamide	44.83 ± 1.07^#^	10.63 ± 0.76^#^	6.75 ± 0.42	36.50 ± 0.45^#^	6.32 ± 0.17
Positive control	64.25 ± 1.11∗	13.52 ± 0.73∗	7.37 ± 0.29	45.49 ± 0.60∗	6.25 ± 0.15
NTU 101 (0.5×)	64.68 ± 1.78∗	12.72 ± 0.45∗	6.80 ± 0.34	46.54 ± 0.24∗	5.85 ± 0.08
NTU 101 (1×)	68.68 ± 2.00∗	14.36 ± 0.97∗	7.79 ± 0.38	45.75 ± 0.61∗	5.92 ± 0.08
NTU 101 (5×)	67.43 ± 2.03∗	14.05 ± 0.70∗	7.37 ± 0.29	47.54 ± 0.59∗	5.65 ± 0.12

1. Data are expressed as the mean ± SD values (n = 8). Statistical analysis was performed using one-way ANOVA with Newman-Keuls post-hoc test. ^#^*p* < 0.05 vs. control group. ∗*p* < 0.05 vs. loperamide group. Positive control: sodium picosulfate 0.52 mg/kg BW; NTU 101 (0.5, 1, and 5×): 1.3, 2.6, and 13 mg/kg BW, respectively.

2. Fecal water content was calculated: fecal water content (%) = (fecal total weight - dry weight/total weight).

### 3.2 Gastrointestinal motility

To assess intestinal peristalsis, the gastrointestinal transit ratio was determined. As shown in Table 2, the transit distance of the activated charcoal meal was significantly higher in the control and NTU 101 (0.5, 1, 5×) treatment groups than in the loperamide group (*p* < 0.05). Gastrointestinal transit ratio was also significantly higher in the control, positive control, and NTU 101 (0.5, 1, 5×) treatment groups than in the loperamide group (*p* < 0.05).

**Table 2:** Total length of the small intestine, transit distance of the charcoal meal, and intestinal charcoal transit ratio of rats.

Groups	Total small intestine length	Transit distance of charcoal meal	Intestinal charcoal transit ratio^a^
	(cm)	(cm)	(%)
Control	141.38 ± 2.54	84.63 ± 3.48	59.86 ± 1.37
Loperamide	141.25 ± 3.60	67.00 ± 2.69^#^	47.43 ± 0.75^#^
Positive control	137.75 ± 1.72	85.63 ± 2.05∗	62.16 ± 1.19∗
NTU 101 (0.5×)	143.25 ± 2.08	85.88 ± 2.62∗	59.95 ± 1.26∗
NTU 101 (1×)	139.13 ± 2.35	84.63 ± 4.12∗	60.83 ± 1.75∗
NTU 101 (5×)	146.63 ± 2.61	91.75 ± 3.30∗	62.57 ± 1.26∗

1. Data are expressed as the mean ± SD values (n = 8). Statistical analysis was performed using one-way ANOVA with Newman-Keuls post-hoc test. #p < 0.05 vs. control group. p < 0.05 vs. loperamide group. Positive control: sodium picosulfate 0.52 mg/kg BW; NTU 101 (0.5, 1, and 5×): 1.3, 2.6, and 13 mg/kg BW, respectivel.

2. Abbreviation meaning of each group is shown in Table 1.

3. Intestinal charcoal transit ratio (%) = (transited distance of charcoal meal/total small intestine length) × 100%.

### 3.3 Intestinal microflora

Compared with the loperamide group, *Bifidobacterium* spp*.* content was significantly higher in the control group (*p* < 0.05), whereas *C. perfringens* content was significantly lower (*p* < 0.05; Table 3). A similar trend was observed for the positive control and NTU 101 (0.5, 1, 5×) treatment groups in comparison with the loperamide group (*p* < 0.05).

**Table 3:** Fecal microflora of rats.

Groups	*Bifidobacterium* spp.	*Clostridium perfringens*
	(log CFU/g)	(log CFU/g)
Control	8.03 ± 0.08	1.91 ± 0.20
Loperamide	7.73 ± 0.05^#^	2.73 ± 0.07^#^
Positive control	8.00 ± 0.09∗	2.13 ± 0.14∗
NTU 101 (0.5×)	8.08 ± 0.07∗	2.10 ± 0.10∗
NTU 101 (1×)	8.09 ± 0.05∗	2.07 ± 0.09∗
NTU 101 (5×)	8.15 ± 0.08∗	1.97 ± 0.14∗

^1^Abbreviation meaning of each group is shown in Table 1.

### 3.4 Analysis of fecal SCFA levels

**Figure 1:**
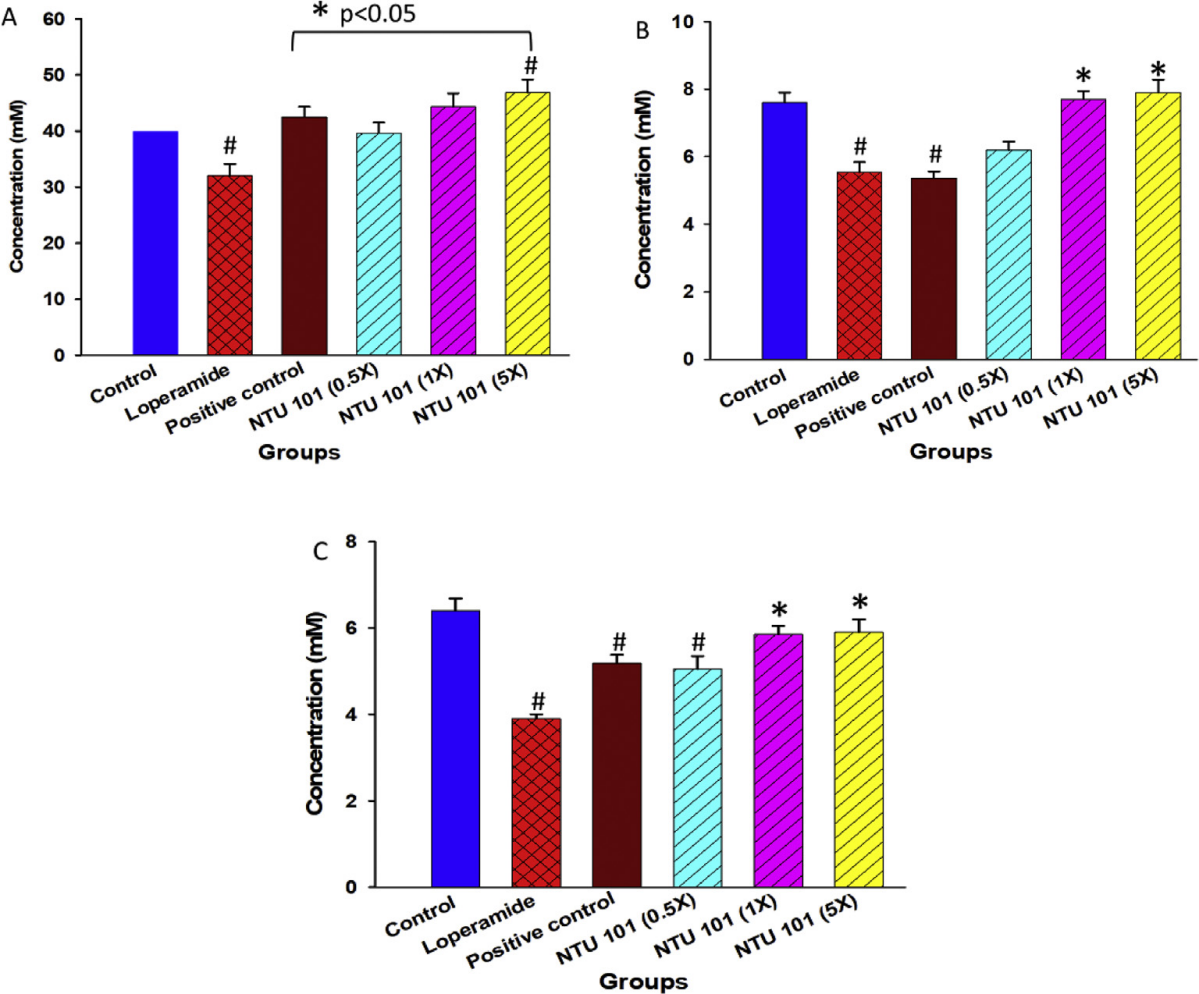
Concentration of short-chain fatty acids (SCFAs) in rat feces. Concentration of acetic (A), propionic (B), and butyric (C) acids. Data are expressed as the mean ± SD values (n = 8). Statistical analysis was performed via one-way ANOVA with Newman-Keuls post-hoc test. ^#^*p* < 0.05 vs. control group. ∗*p* < 0.05 vs. loperamide group.

Fecal SCFA levels, namely acetic, propionic, and butyric acid, are shown in Figures 1A-1C. Compared with the loperamide group, fecal SCFA levels were significantly higher in the control, positive control (*p* < 0.05) and NTU 101 (1× and 5×) treatment groups (*p* < 0.05).

### 3.5 Histopathologic examination of rat colons and colonic mucosal layer thickness

**Figure 2:**
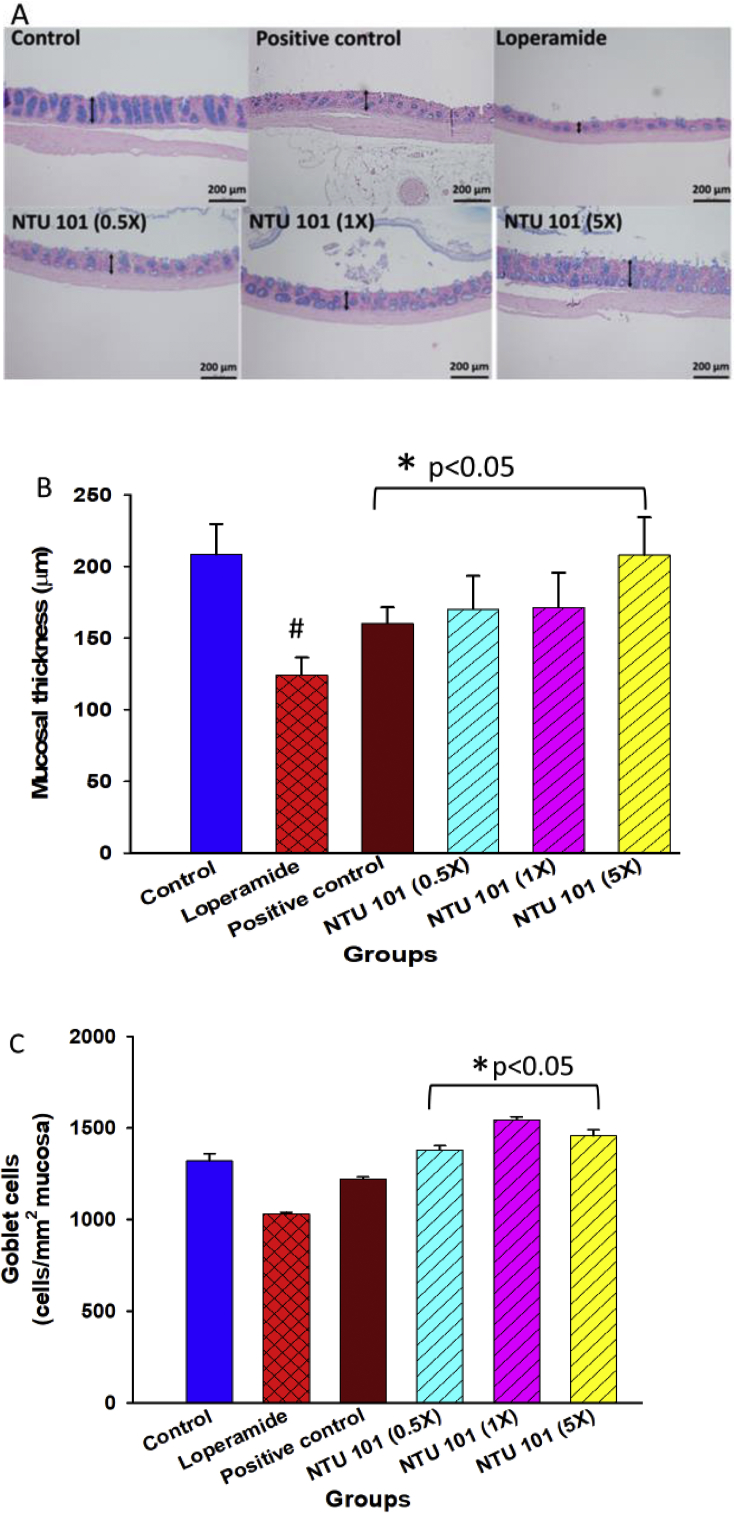
Histopathological examination of rat colons (A), colonic mucosal layer thickness (B), and colonic mucus-producing goblet cells (C). ^1^Abbreviation meaning of each group is shown in Figure 1.

Goblet cells secrete mucus, a viscous fluid composed primarily of highly glycosylated proteins called mucins suspended in a solution of electrolytes. Surface colonic goblet cells continuously secrete mucus to maintain the inner mucus layer, whereas goblet cells of the colonic and small intestinal crypts secrete mucus upon stimulation, e.g., after endocytosis or in response to acetyl choline (Birchenough et al., 2015). We found that the colonic mucosa was significantly thicker in the control, positive control, and NTU 101 treatment groups than in the loperamide group (Figures 2A and 2B). The number of mucus-containing goblet cells was not significantly different between the loperamide group and the control group; however, it was significantly higher in the NTU 101 treatment groups than in the loperamide group (Figure 2C).

### 3.6 c-Kit immunohistochemistry staining of interstitial cells of Cajal (ICC) and histopathology of colon inflammation

**Figure 3:**
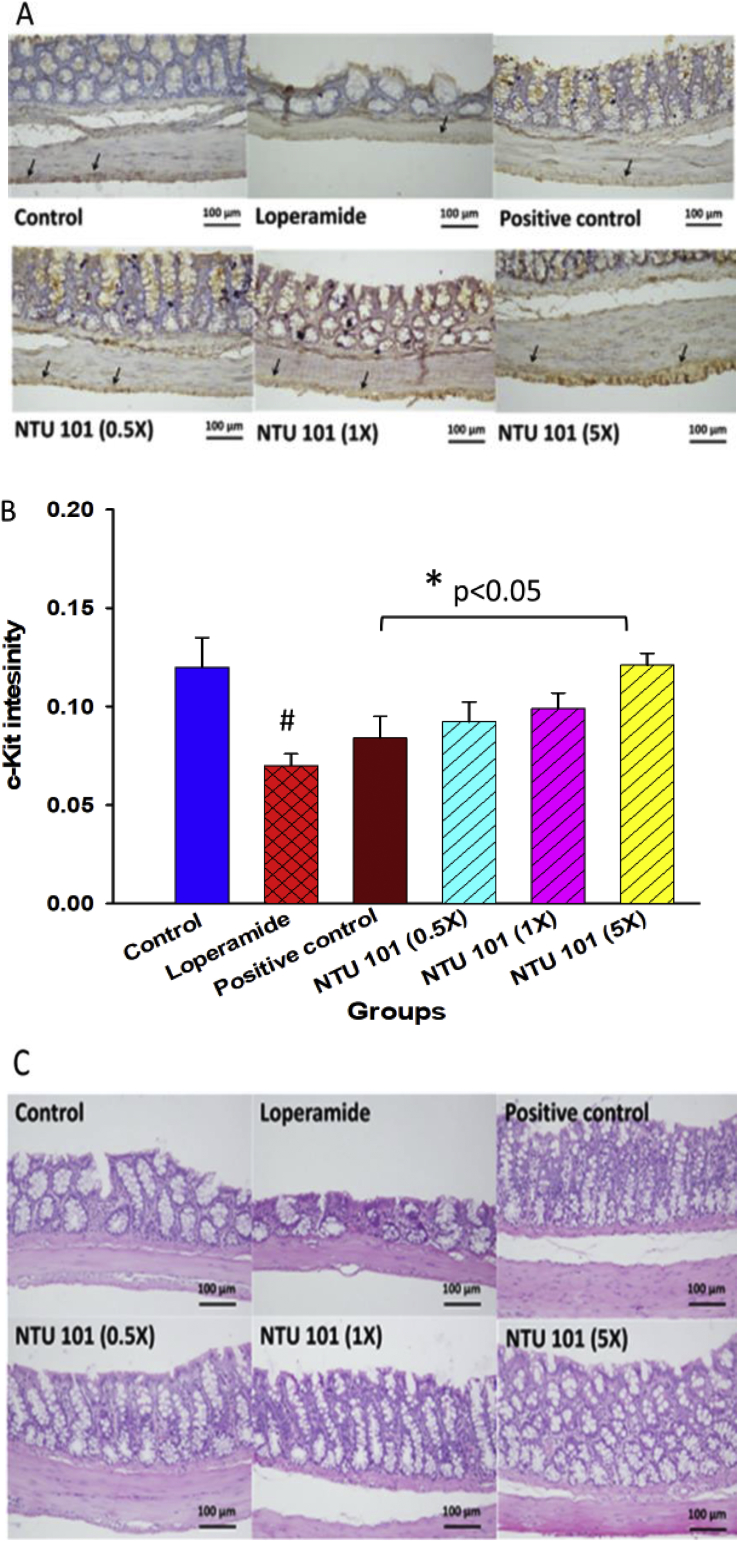
c-Kit immunohistochemical staining of intestinal cells of Cajal (ICCs) (A), c-Kit staining intensity in the colon (B), and hematoxylin and eosin staining of the colon (C). ^1^Abbreviation meaning of each group is shown in Figure 1. ^2^ c-Kit: tyrosine protein kinase kit (CD117). The arrows indicate ICCs.

ICCs were initially identified by Cajal who first described nerve-like cells at the ends of motor neurons in organs innervated by peripheral nerves (Bansil and Turner, 2018). ICCs are typically identified by their ultrastructure and expression of tyrosine protein kinase (c-Kit; CD 117), and are found in the muscle layer of the gastrointestinal tract. Immunohistochemistry staining for c-Kit was performed and immunoreactive cells in the muscularis externa of the intestinal wall were identified as ICCs and mast cells. ICCs are essential for normal digestive tract function, both as pacemakers and as intermediates between nerves and smooth muscle cells. ICCs located in the myenteric plexus (ICC-MY)—considered to be the main functioning cells—were stained as shown in Figure 3A. The staining intensity was then quantified (Figure 3B) and the results showed that ICC performance in the constipation-induced group was significantly lower than that of the control group (p < 0.05). NTU 101 treatment significantly improved ICC performance (p < 0.05). Gastrointestinal motility function and its regulation is a complex process involving collaboration and communication between multiple cell types, such as enteric neurons, ICCs, and smooth muscle cells (Al-Shboul, 2013). The loperamide-induced constipation model was used to evaluate NTU 101 efficacy in improving constipation. H&E staining was used to examine inflammation in the intestine; however, Figure 3C shows that there was no infiltration of inflammatory cells in the colon of NTU 101 treatment groups.

## 4 Discussion

Loperamide-induced delay in colonic transit occurs due to inhibition of stool frequency and increased colonic contractions in humans (Kojima et al., 2009). This drug inhibits intestinal water secretion and colonic peristalsis (Hughes et al., 1984), which extends the fecal evacuation time and delays intestinal luminal transit (Yamada and Onoda, 1993). Thus, loperamide-induced constipation is considered to be a model of spastic constipation (Takasaki et al., 1994). We employed a loperamide-induced rat model of constipation, characterized by decreased fecal pellets, fecal water content (Ohashi et al., 2001; Wintola et al., 2010; Ashafa et al., 2011), gastrointestinal transit ratio (Wintola et al., 2010), and fecal SCFA levels (Li et al., 2015), accompanied by an imbalance in intestinal microflora (Chen et al., 2010), and ICC (Li et al., 2015). The rats were then fed NTU 101 lyophilized powder to evaluate its efficacy in managing constipation. Improvements in constipation were assessed by examining the intestinal conditions of the rats. Loperamide treatment decreased in the fecal pellet number and water content (Table 1). We found that NTU 101 increased the number of mucus-containing goblet cells, thereby enhancing bowel movements and thickening the colonic mucosa, which resulted in promotion of cell growth and differentiation by SCFAs, further increasing the number of ICCs (Figures 1 and 2). Inflammatory cell infiltration was not observed in the colon (Figures 2 and 3); however, the aforementioned conditions in constipated rats were improved after the rats were fed NTU 101 lyophilized powder.

Previous studies have reported that LAB improves constipation, although the underlying mechanisms remain unclear. NTU 101 is a LAB strain with lactic acid as its major fermentation product. LAB can provide lactate to other intestinal microflora, which can transfer lactate to SCFAs or synthesize butyric acid from acetic acid (Ohashi et al., 2001; Duncan et al., 2002; Tsukahara et al., 2006). NTU 101 can enhance the content of *Bifidobacterium* spp., because the main fermentation metabolite of NTU 101 is lactic acid, which can be used as a substrate for other enteric bacteria that can metabolize lactic acid. *Bifidobacterium* are used for production of SCFAs or synthesis of butyric acid from acetic acid (Gomes and Malcata, 1999). Increased *Bifidobacteria* and *Lactobacillus* counts in feces improve the ratio of beneficial to harmful bacteria in the gut, and are involved in increasing the proliferation of beneficial bacteria in the body, thereby improving gut flora. The results of clinical studies indicate that probiotics could alter the composition of gut flora to help humans resist various pathogens and inhibit harmful bacteria, restore the balance of gut flora, and enhance the defensive capability of the digestive tract. In addition to its effects on host digestive function, gut flora also affects other physiological functions, particularly the immune system (Bengmark, 2013; Hooper et al., 2012; Thursby and Juge. 2017). Accordingly, we speculated that fecal pH values were lower in the NTU 101 groups than in the loperamide group owing to lactate production in the NTU 101 groups or SCFA production by the intestinal microflora after fermentation. SCFAs (e.g., acetic acid, propionic acid, and butyric acid) are produced from polysaccharides, oligosaccharides, and proteins by the intestinal microflora (MacFarlane and MacFarlane, 2003). SCFAs are nutrients for colonic epithelial cells and can modulate colonic pH value, cell volume, cell proliferation, and differentiation as well as regulate gene expression (Cook and Sellin, 1998). Moreover, reduction in intestinal pH value resulting from an increase in SCFA levels indirectly influences the composition of intestinal microflora (Hijova and Chmelarova, 2007; Duncan et al., 2009). SCFAs also promote intestinal motility (Fukumoto et al., 2003) and stimulate mucus secretion (Shimotoyodome et al., 2000b; Gaudier et al., 2004).

In the present study, the positive control group displayed improvement in intestinal microflora. The positive control group received a laxative (sodium picosulfate), which was hydrolyzed by intestinal bacteria to the active metabolite 4,4ʹ-dihydroxydiphenyl-(2-pyridiyl)-methane, stimulating intestinal peristalsis and improving bowel movement (Hoy et al., 2009). Therefore, we speculated that laxatives promote intestinal motility instead of reducing the colonic retention time of stools, thereby decreasing the retention time of harmful substances in the colon and regulating the intestinal bacterial imbalance resulting from constipation. The mechanism of action of NTU 101 may thus be similar to that of a laxative.

**Figure 4:**
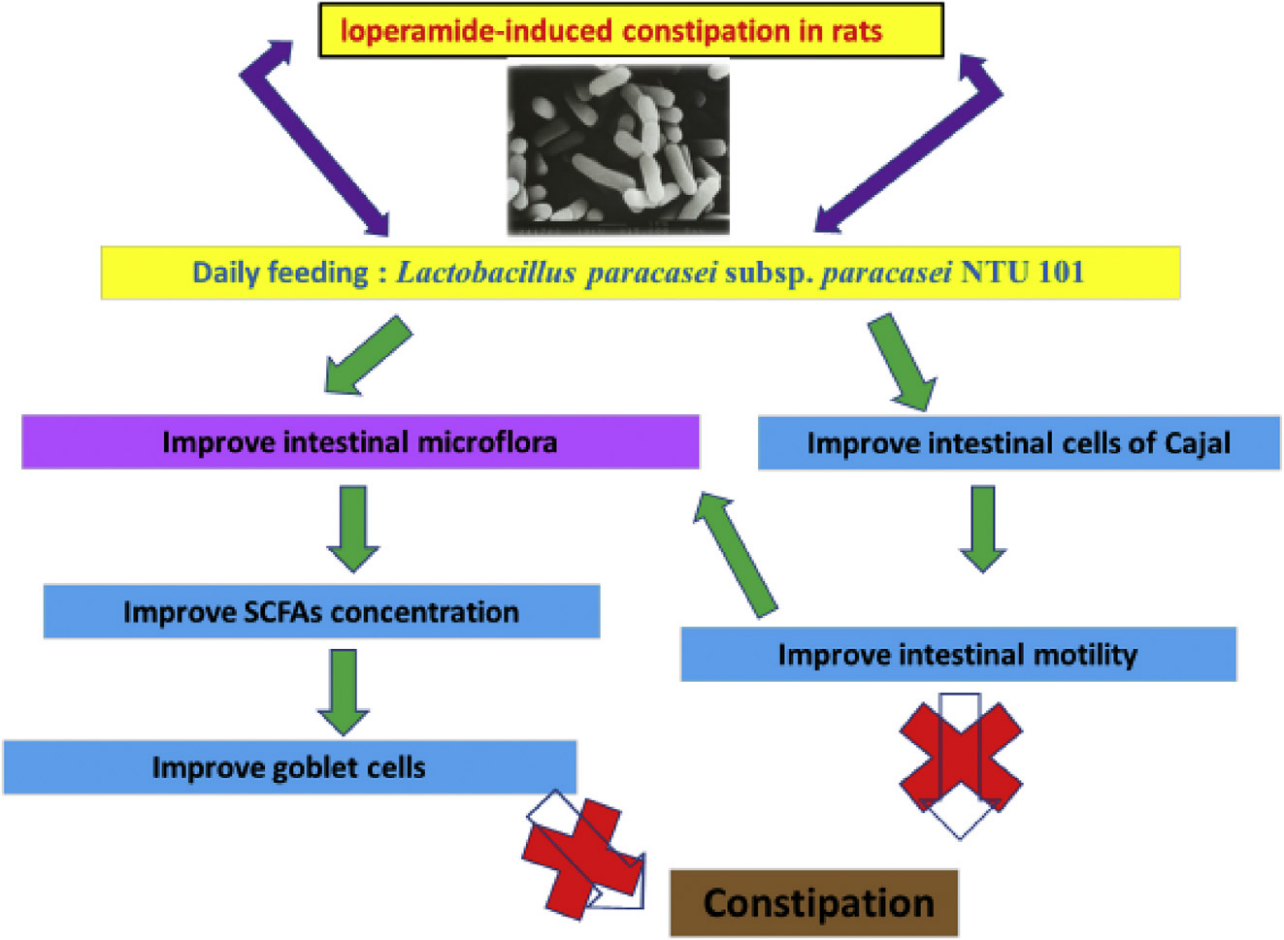
Potential mechanism underlying the improvements in constipation mediated by *Lactobacillus paracasei* subsp. *paracasei* NTU 101 lyophilized powder.

In summary, NTU 101 can exert a treatment effect on certain types of constipation such as that induced by the opioid drug loperamide. NTU 101 lyophilized powder increased the number of fecal pellets and fecal water content, promoted intestinal motility, and reduced colonic fecal retention. In addition, it increased the number of beneficial bacteria (*Bifidobacterium* spp.) and reduced the number of harmful bacteria (*C. perfringens*), thereby regulating intestinal flora. Furthermore, NTU 101 increased the fecal SCFA levels and reduced fecal pH value, thus improving intestinal conditions and mucosal thickness, which was accompanied by an improvement in intestinal function, increased number of goblet cells that lubricated the intestinal tract to aid defecation, and enhancement of ICC performance, indicating an improvement in bowel movements. The underlying mechanism thus involves improvement in intestinal constipation through SCFAs and ICCs (Figure 4).

## Declarations

### Author contribution statement

Chien-Li Chen: Performed the experiments; Wrote the paper.

Sih-Han Chao: Performed the experiments.

Tzu-Ming Pan: Conceived and designed the experiments; Analyzed and interpreted the data; Contributed reagents, materials, analysis tools or data; Wrote the paper.

### Funding statement

This research did not receive any specific grant from funding agencies in the public, commercial, or not-for-profit sectors.

### Competing interest statement

The authors declare no conflict of interest.

### Additional information

No additional information is available for this paper.
